# Long-term exposure to BAY2416964 reduces proliferation, migration and recapitulates transcriptional changes induced by AHR loss in PyMT-induced mammary tumor cells

**DOI:** 10.3389/fonc.2024.1466658

**Published:** 2024-10-10

**Authors:** Ninni Elise Olafsen, Siddhartha Das, Chiara Gorrini, Jason Matthews

**Affiliations:** ^1^ Department of Nutrition, Institute of Basic Medical Sciences, Faculty of Medicine, University of Oslo, Oslo, Norway; ^2^ School of Molecular and Cellular Biology, University of Leeds, Leeds, United Kingdom; ^3^ Princess Margaret Cancer Centre, University Health Network, Toronto, ON, Canada; ^4^ Department of Pharmacology and Toxicology, University of Toronto, Toronto, ON, Canada

**Keywords:** aryl hydrocarbon receptor, BAY2416964, breast cancer, PyMT, GNF351, proliferation, kynurenine

## Abstract

The aryl hydrocarbon receptor (AHR) is a ligand activated transcription factor which in certain cancer types drives pro-survival processes that facilitate tumorigenesis, malignant cell migration, invasion, and metastasis. Much of AHR’s pro-tumorigenic action is due to its activation by the oncometabolite, kynurenine. Because of this AHR antagonists are being actively investigated as new anti-tumor therapy. In this study we compared the effects of treatment with the AHR antagonists, BAY2416964 and GNF351, to that of AHR knockout in PyMT murine mammary cancer cells. BAY2416964 and GNF351 effectively inhibited kynurenine-dependent increases in *Cyp1a1* and *Cyp1b1* mRNA levels. CRISPR/Cas9-generated PyMT Ahr^KO^ cells exhibited reduced cell proliferation compared with controls, but treatment with 1 μM BAY2416964 for 96 h had no effect on the proliferation of wildtype cells. To further examine the differences between AHR knockout and short term BAY2416964, we generated long-term BAY2416964 (LT-BAY) cells by exposing wildtype cells to 1 μM BAY2416964 for at least 6 weeks. Similar to Ahr^KO^ cells, LT-BAY cells exhibited reduced cell proliferation and migration compared with wildtype cells. No differentially expressed genes (DEGs) were identified in wildtype cells exposed to 1 μM BAY2416964 for 24 h; however, 46.4% of DEGs overlapped between Ahr^KO^ and LT-BAY cells including gene regulated cell proliferation. Our data reveal long-term pharmacological inhibition of AHR by BAY2416964 closely resembles AHR loss in a mouse model of breast cancer.

## 1 Introduction

The aryl hydrocarbon receptor (AHR) is a ligand activated transcription factor and member of the basic helix-loop-helix-PER-ARNT-SIM (bHLH-PAS) family. AHR was initially identified as a key regulator in mediating the toxicity of environmental contaminants, such as 2,3,7,8-tetrachlorodibenzo-p-dioxin (TCDD; dioxin) and benzo[*a*]pyrene (1). AHR is, however, also activated by many dietary compounds, microbiota-derived indoles and endogenous compounds including, catabolic metabolites of tryptophan, such as kynurenic acid and kynurenine (2, 3). In the absence of ligand, AHR is in the cytosol in a complex with heat shock protein 90 (Hsp90), immunophilin-like protein hepatitis B virus X-associated protein 2 (XAP2) and the co-chaperone p23. Upon ligand binding, AHR translocates into the nucleus where it heterodimerizes with AHR nuclear translocator (ARNT). This heterodimer complex binds to AHR response elements (AHREs; also known as dioxin response elements (DREs)) located in regulatory regions of its target genes, such as cytochrome P450 1A1 (CYP1A1), CYP1B1, AHR repressor (AHRR), and poly-ADP-ribose polymerase 7 (PARP7; also known as TCDD-inducible poly-ADP-ribose polymerase (TIPARP)) (4, 5). AHR activity is negatively regulated by ligand-induced proteolytic degradation of the AHR protein, enzymatic degradation of its ligands by CYP450 enzymes, and through two negative feedback loops. One negative feedback loop includes AHRR-mediated inhibition of AHR and ARNT heterodimerization, while the other includes the mono-ADP ribosylation of AHR by PARP7 (5, 6).

Multiple lines of evidence support a key role for AHR in cellular homeostasis, energy metabolism, immune cell responses, inflammation and cancer (7–9). AHR is upregulated and constitutively active in many different cancer types, including neck squamous cell carcinoma, non-small cell lung cancer, colorectal cancer, and breast cancer (10, 11). AHR regulates multiple stages of tumorigenesis, including cell proliferation, angiogenesis, tissue invasion, tumor-associated inflammation, and metastasis (4). Whether overexpression of AHR is a positive or negative prognostic factor for cancer patient prognosis depends on the tumor type. This is because AHR exhibits both pro- and anti-tumorigenic effects which are ligand-, cell- and tumor type-dependent. AHR acts as a tumor suppressor in models of colon cancer but promotes the growth and invasiveness of gastric cancer cells (12, 13). In breast cancer, AHR activation reduces the proliferation of estrogen receptor-positive breast cancer cells, while both AHR agonists and antagonists have been reported to reduce colony formation, migration and metastasis of triple negative breast cancer cell lines (14, 15). These data reveal the complexity of targeting AHR activity in cancer and suggest that depending on the tumor type, both AHR agonists and antagonists could be useful cancer therapeutics.

Recent studies have reported that endogenous AHR signaling in barrier organs and in tumors is tightly linked to tryptophan metabolism (16). Tryptophan through its degradation by indoleamine-2,3-dioxygenase 1 (IDO1) and tryptophan-2,3-dioxygenase 2 (TDO2) to kynurenine has emerged as an important metabolic regulator of tumor progression, immune suppression and immune tolerance in the tumor microenvironment. These effects of kynurenine are mediated by the activation of AHR resulting in the recruitment of immunosuppressive tumor associated macrophages and T regulatory cells (17). In this feed forward mechanism, AHR upregulates IDO1 leading to increased kynurenine levels, which results in AHR-dependent increases in the levels of immune checkpoint inhibitor proteins, programmed death receptor (PD1) and its ligand, PDL1, promoting immune suppression and enhancing tumorigenicity (18).

AHR’s autonomous role in tumorigenesis and cancer progression, combined with its immune suppression properties suggest that inhibition of AHR offers an antitumor therapeutic option to reduce tumor growth and help release the “brake” that cancer uses to evade the immune system. Because of this, several AHR antagonists, such as, GNF351, CH223191, StemRegenin 1, IK-175 and BAY2416964 have been described (19–22). IK-175 was reported to exhibit antitumor activity in syngeneic mouse models of colorectal cancer and melanoma both as monotherapy and when combined with checkpoint inhibition treatment (22). More recently it was shown that BAY2416964 restores immune cell function and enhances antigen-specific cytotoxic T cell responses *in vitro*, but also promotes a proinflammatory tumor microenvironment resulting in reduced tumor growth in mice (21).

Despite the promising anticancer effects of BAY2416964 by promoting a proinflammatory tumor microenvironment, there are relatively few studies characterizing its ability to inhibit cancer cell signaling and proliferation. Here we compared the effect of BAY2416964 treatment with AHR loss on cell growth, invasion and gene expression in PyMT mouse mammary cancer cells. We included studies with short-term (<24 h) treatment with BAY2416964 and long-term treatment of at least 6 weeks. Since clinical treatment courses often take months, the long-term BAY2416964 treatment will provide valuable insight into how extended treatment affects cancer cell signaling if it becomes an approved therapeutic. BAY2416964 inhibited kynurenine-induced activation of AHR after 6 h and after long-term treatment of at least 6 weeks. The reduced cell proliferation and migration observed after AHR knockout were only observed in long-term BAY2416964 (LT-BAY) treated cells. RNA-sequencing studies revealed that 46.4% of the differentially expressed genes (DEGs) overlapped between LT-BAY and Ahr^KO^ cells. Our findings show that prolonged pharmacological inhibition of AHR with BAY2416964 mimics many, but not all, of the phenotypes observed after *Ahr* knockout.

## 2 Methods and materials

### 2.1 Chemicals and reagents

Dimethyl sulfoxide (DMSO) and L-kynurenine were purchased from Sigma-Aldrich (St. Louis, MO, USA). BAY2416964 and GNF351 were purchased from MedChemExpress (Monmouth Junction, NJ, USA). All ligands were stored according to the manufacturer’s instructions.

### 2.2 Cell lines and cell culturing

The PyMT cell line was isolated from a spontaneous mammary tumor that developed in mouse mammary tumor virus-polyoma virus middle T-antigen (MMTV-PyMT) female mice on a C57BL/6 background as previously described (23). Briefly, after harvesting and isolation of the PyMT cancer cells, the cells were cultured in Dulbecco’s Modified Eagle Media (DMEM)/F12 medium (Sigma-Aldrich) containing 10% v/v fetal bovine serum (FBS; Sigma-Aldrich), 1% v/v l-glutamine (Sigma-Aldrich), 1 µg/ml hydrocortisone (Sigma-Aldrich), 5 µg/ml insulin (Sigma-Aldrich), and 5 ng/ml epidermal growth factor (EGF; Sigma-Aldrich). The CR705 cell line were isolated from spontaneous pancreatic tumors of the LSL-Kras^G12D/+^;LSL-Trp53^R172H/+^;Pdx-1-Cre (KPC) mice (24). For all experiments, PyMT cells were cultured in DMEM (Sigma-Aldrich) and CR705 cells in RPMI medium, supplemented with 10% v/v FBS (Sigma-Aldrich), 1% v/v penicillin-streptomycin (Sigma-Aldrich), and 1% v/v l-glutamine (Sigma-Aldrich). Cells were incubated at 37°C and 5% CO_2_ in a humidified environment and sub-cultured when they reached 80% confluency.

### 2.3 Generation of the PyMT Ahr^KO^ cell line

PyMT Ahr^KO^ cells were generated using CRISPR/Cas9. Briefly, the guide oligos forward 5´-AAACTCTAAGCGACACAGAGACCGC-3´ and reverse 5´-CACCGCGGTCTCTGTGTCGCTTAGA-3´ were annealed and ligated into the pSpCas9(BB)-2A-Puro (PX459) plasmid (Addgene, Watertown, MA, USA; plasmid #62988). PyMT cells were transfected with 4 µg of PX459 containing AHR gRNA. Forty-eight hours after transfection, cells were selected with 4 µg/ml puromycin for 5 days. PyMT PX459 cells which represent a pooled cell population were expanded. For the PyMT cells transfected with PX459 AHR gRNA we did single cell dilution cloning and isolated independent clones as previously described (25). After screening the clones for AHR activity, DNA was isolated, and the region surrounding the target site of the *Ahr* gene was amplified using forward: 5´-TGTTTCGTCGGTAGAGCAGT-3´ and reverse 5´-AGTCCTAGCCCCAATCAGTCT-3´. The presence of indels resulting in frame shift mutations was confirmed by DNA sequencing. PyMT cells transfected with PX459 vector with no gRNA and exposed to puromycin as described for Ahr^KO^ cells, were used as control cells in further experiments and will be referred to as WT cells. In addition, we generated long-term-BAY2416964 (LT-BAY) cells by supplementing the media of WT cells with 1 µM of BAY2416964 for at least 6 weeks.

### 2.4 RT-qPCR

PyMT cells were plated at a density of 5×10^4^ cells/well in a 24-well plate and treated with test compounds the following day. RNA was isolated using the Aurum™ Total RNA isolation kit (BioRad, Hercules, CA, USA) according to manufacturer’s instructions. RNA was converted to cDNA using High-Capacity cDNA Reverse Transcription kit (Applied Biosystems, Waltham, MA, USA), and qPCR was performed using the SsoAdvanced™ Universal SYBR^®^ Green Supermix (BioRad). Primers used were *Tbp* forward 5´-GCACAGGAGCCAAGAGTGAA-3´, reverse 5´-TAGCTGGGAAGCCCAACTTC-3´, *Cyp1a1* forward 5´-CGTTATGACCATGATGACCAAGA-3´, reverse 5´-TCCCCAAACTCATTGCTCAGAT-3´, *Cyp1b1* forward 5´-CCAGATCCCGCTGCTCTACA-3´, reverse 5´-TGGACTGTCTGCACTAAGGCTG-3´, *Parp7* forward 5´-TCCCCGTGTCTGTGGAAAGCATG-3´, reverse 5´-TTGACCGGAGGGGGCCTTCT-3´, *Cdh2* forward 5´-AGCGCAGTCTTACCGAAGG-3´, reverse 5´-TCGCTGCTTTCATACTGAACTTT-3´, *Grb10* forward 5´-GGTGAAAGAGGTAGGACGCAAG-3´, reverse 5´-GATGCTGCTTTCTTCCAGGTCAG-3´, *Gas1* forward 5´-AGATGGTCGGGAACACTGAC-3´, reverse 5´-CCTCAACATCCCTTCTCTCCAA-3´, *Mmp2* forward 5´-CAAGGATGGACTCCTGGCACAT-3´, reverse 5´-TACTCGCCATCAGCGTTCCCAT-3´.

### 2.5 Western blotting

PyMT cells were plated in a 6-well plate at a density of 2×10^5^ cells/well and incubated with test compounds for 6 h before harvesting. The cells were harvested and lysed using 10 mM Tris-HCl buffer containing 1 mM EDTA and 1% SDS, with pH of 8.0. Samples were sonicated at high intensity for 2 × 30 seconds on/off using a Bioruptor (Diagenode, Denville, NJ, USA). Cytoplasmic and nuclear fractions were harvested using the NE-PER™ Nuclear and Cytoplasmic Extraction Reagents kit (Thermo Fischer Scientific, Waltham, MA, USA) according to manufacturer’s instructions. The protein concentration was measured using Pierce™ BCA Protein Assay Kit (Thermo Fischer Scientific). Proteins were separated on 4-20% SDS-PAGE gels (BioRad) and transferred to polyvinylidene fluoride membranes (Millipore, Burlington, MA, USA). The membranes were incubated with primary antibodies dissolved in 5% skim milk over night at 4°C, followed by appropriate secondary antibodies for 1 h at room temperature. Antibodies used were anti-AHR (bml-sa210-0100; Enzo Life Sciences, Farmingdale, NY, USA), anti-PARP1 (sc-7150; Santa Cruz, Dallas, TX, USA), anti-CASPASE-3 (9662S; Cell Signaling Technology, Danvers, MA, USA), anti-Lamin A/C (2032S; Cell Signaling Technology), anti-Tubulin (T5168; Sigma-Aldrich) and anti-β-actin (AC-74; Sigma-Aldrich). Protein bands were visualized with SuperSignal™ West PICO or DURA extended Duration Substrate (Thermo Fischer Scientific).

### 2.6 Ectopic AHR expression in Ahr^KO^ cells

For mRNA expression experiments, PyMT Ahr^KO^ cells were plated in a 12-well plate at a density of 2.5×10^4^ cells/well and incubated 24 h. The cells were then transfected with 500 ng of pcDNA3.1-*Ahr* (mouse *Ahr^b1^*) plasmid, or empty plasmid, using Lipofectamine™ 3000 (Thermo Fischer Scientific) according to manufacturer’s instructions. The cells incubated 48 h or 72 h. RT-qPCR was performed as described above. For protein, the cells were plated at a density of 1 × 10^5^ cells/well in a 6-well plate and transfected the following day with 2.5 µg of pcDNA3.1-Ahr or empty plasmid using Lipofectamine 3000. Protein expression was determined as described in section 2.5.

### 2.7 Proliferation assay

Cell proliferation was determined using an IncuCyte instrument. Briefly, PyMT cells were plated in a 96-well plate at a cell density of 5×10^2^ cells/well in 100 µl media. After 24 h incubation the cells were treated with test compounds and placed in an IncuCyte instrument (Sartorius, Göttingen, Germany). Cell confluency was measured every 6 h for 96 h.

### 2.8 Transwell migration assay using the xCELLigence instrument

The migratory potential of the different PyMT cells were measured with a transwell migration assay using the Agilent CIM-plate 16 for the xCELLigence instrument (Agilent Technologies, Santa Clara, CA, USA) according to manufacturer’s instructions. Briefly, 160 µl media containing 10% FBS was added to each well of the lower chamber, and 50 µl FBS-free media to the wells of the upper chamber and incubated in a 37°C incubator for 1 h for the membranes to reach equilibrium. Subsequently, cells were trypsinized and centrifuged at 200xg for 5 min to remove media and washed twice with FBS-free media. Cells were then counted and 6×10^4^ cells/well were plated in 100 µl FBS-free media in the upper chamber of the plates. Migration was measured every 15 min for 8 h.

### 2.9 Cell toxicity assay

Cells were plated in a 96-well plate at a density of 2.5×10^3^ cells/well and dosed with ligands the next day for 6 h. CellTiter-Glo^®^ Luminescent Cell viability Assay (Promega, Madison, WI, USA) was used according to manufacturer’s instructions to measure ATP levels at baseline and at the end of experiment.

### 2.10 RNA sequencing

PyMT WT, Ahr^KO^ and LT-BAY cells were plated in a 12-well plate at a density of 1×10^5^ cells/well in quadruplicates. Total RNA was isolated with the Aurum™ Total RNA isolation kit (BioRad), following manufacturer’s protocol. The raw RNA seq paired end fastq files were quantified using the Salmon tool (26) with “libtype” flag as automatic and mm10 version of the Salmon index file. Salmon carries out a pseudoalignment on the fastq files to quantify the transcript abundance from RNA sequencing. The index was generated using the salmon “index” flag with the mm10 transcripts fasta file supplied. The “tximport” import function from the tximport package (v1.26.1 (27);) was used to import the Salmon quantification data for further processing including differential expression analysis by DESeq2 (28). For all comparisons, PyMT WT samples were considered as the control. Significant genes were considered as those with absolute log fold change greater than 1 and Benjamin Hochberg false discovery rate value of differential expression less than 0.01 and tested using the Wald Test implemented in DESeq2. Pathway analysis was done using Ingenuity pathway analysis (Qiagen, Hilden, Germany).

### 2.11 Statistical analyses

All data are presented as mean and standard error of the mean (S.E.M) of three independent experiments if not specified otherwise. All statistical analyses were done using Graphpad Prism 9.3.1 (GraphPad Software, Boston, MA, USA). Significant differences were identified by Student’s t-test, one-way analysis of variance (ANOVA), two-way ANOVA, and area under the curve. Significant differences were set to *p*<0.05.

## 3 Results

### 3.1 BAY2416964 and GNF351 inhibit kynurenine-induced AHR activation in PyMT mouse mammary cancer cells

**Figure 1:**
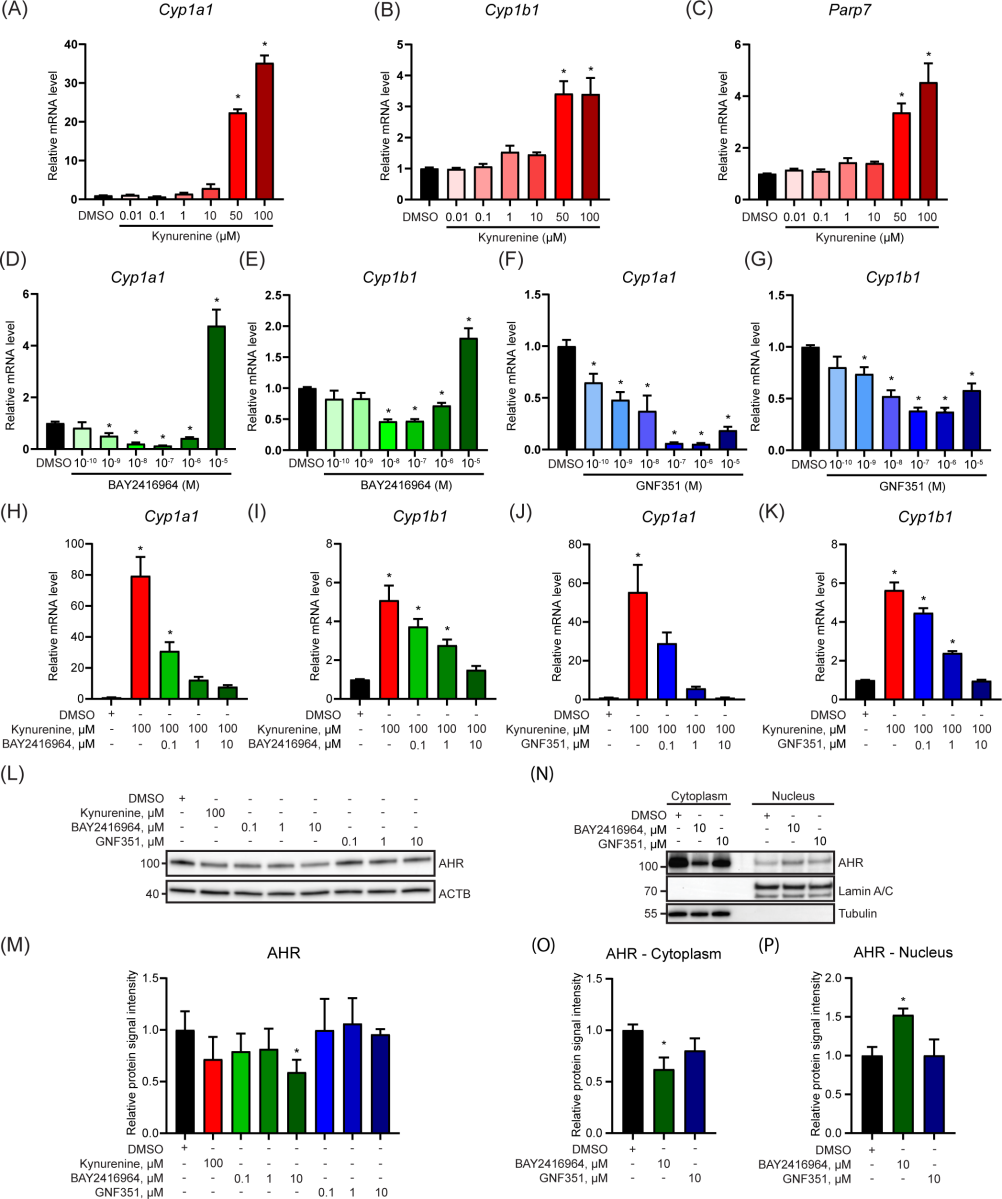
BAY2416964 inhibits kynurenine-induced AHR activity in PyMT mouse mammary cancer cells. Kynurenine increased *Cyp1a1* **(A)**, *Cyp1b1* **(B)** and *Parp7* **(C)** mRNA levels in a dose-response manner in PyMT cells as measured by RT-qPCR. Relative mRNA levels of *Cyp1a1* **(D)** and *Cyp1b1* **(E)** after dose-response treatment with BAY2416964. *Cyp1a1* **(F)** and *Cyp1b1* **(G)** mRNA levels after treatment with increasing doses of GNF351. Relative *Cyp1a1* and *Cyp1b1* mRNA levels after treatment with increasing amounts of BAY2416964 **(H, I)** or GNF351 **(J, K)** in the presence of 100 µM kynurenine. RT-qPCR results are generated by samples treated 6 h with test compounds and presented as mean ± S.E.M n=3. **(L)** Western blot of PyMT cells treated for 6 h with kynurenine, BAY2416964 or GNF351 at the concentrations indicated. Representative image n=3. **(M)** Quantification of western blot. **(N)** AHR protein in cytoplasmic and nuclear fractions after 6 h treatment with BAY2416964 or GNF351. Representative image n=3. **(O)** Protein quantification of cytoplasmic AHR relative to loading control (Tubulin). **(P)** Protein quantification of nuclear AHR relative to loading control (Lamin A/C). *p<0.05 compared with control (DMSO).

Since AHR protein levels are increased in human breast carcinoma cell lines compared with primary human mammary epithelial cells (11), and there is emerging interest in inhibiting AHR to treat cancer, we compared the ability of two AHR antagonists, BAY2416964 and GNF351, to inhibit AHR signaling in a PyMT mouse mammary cancer cell line. BAY2416964 is a newly developed AHR antagonist for which there is limited knowledge of its ability to inhibit AHR activity and affect breast cancer cell proliferation (21), while GNF351 is well-known pure AHR antagonist (19). PyMT cells were isolated from a spontaneous mammary tumor that developed in a MMTV-PyMT mouse, which is commonly used as a model of luminal B breast cancer (29). To verify the AHR responsiveness of PyMT cells we exposed them to increasing concentrations of kynurenine (0.01-100 μM) and determined *Cyp1a1*, *Cyp1b1* and *Parp7* mRNA levels. Significant increases in the expression levels of all three genes were only observed at 50 μM and 100 μM kynurenine (**Figures 1A–C**). Notably, these concentrations are well beyond the reported physiological levels of kynurenine which range from 1-2 μM (30). We next exposed cells to increasing concentrations of BAY2416964 or GNF351. Treatment with 0.001-1 μM BAY2416964 reduced *Cyp1a1*, and 0.01-1 μM also reduced *Cyp1b1* mRNA levels compared with DMSO. Conversely, 10 µM BAY2416964 significantly increased *Cyp1a1* and *Cyp1b1* mRNA levels (**Figures 1D, E**). GNF351 reduced *Cyp1a1* mRNAs levels at all doses tested, while doses of 0.001-10 μM reduced *Cyp1b1* mRNA levels compared with DMSO control (**Figures 1F, G**). Because CYP1A1 levels can be increased as a result of cellular stress, reactive oxygen species, and cytotoxicity (31), we determined if higher concentrations of BAY2416964 and GNF351 induced apoptosis or caused cytotoxicity. We did not observe cleavage of CASPASE-3 or PARP1, suggesting that the cells were not undergoing apoptosis (**Supplementary Figure S1A**). We also did not find any evidence of cytotoxicity, indicating that the observed increase in *Cyp1a1* was not due to cell stress (**Supplementary Figure S1B**). BAY2416964 or GNF351 dose-dependently repressed kynurenine-induced increases in *Cyp1a1* and *Cyp1b1* mRNA levels (**Figures 1H–K**). We then determined the AHR protein levels after treatment with kynurenine, BAY2416964 or GNF351. Kynurenine did not affect AHR protein levels, whereas 10 µM BAY2416964 but not 10 µM GNF351 decreased AHR protein levels (**Figures 1L, M**). BAY2416964 but not GNF351 also increased nuclear translocation of AHR (**Figures 1N–P**).

**Figure 2:**
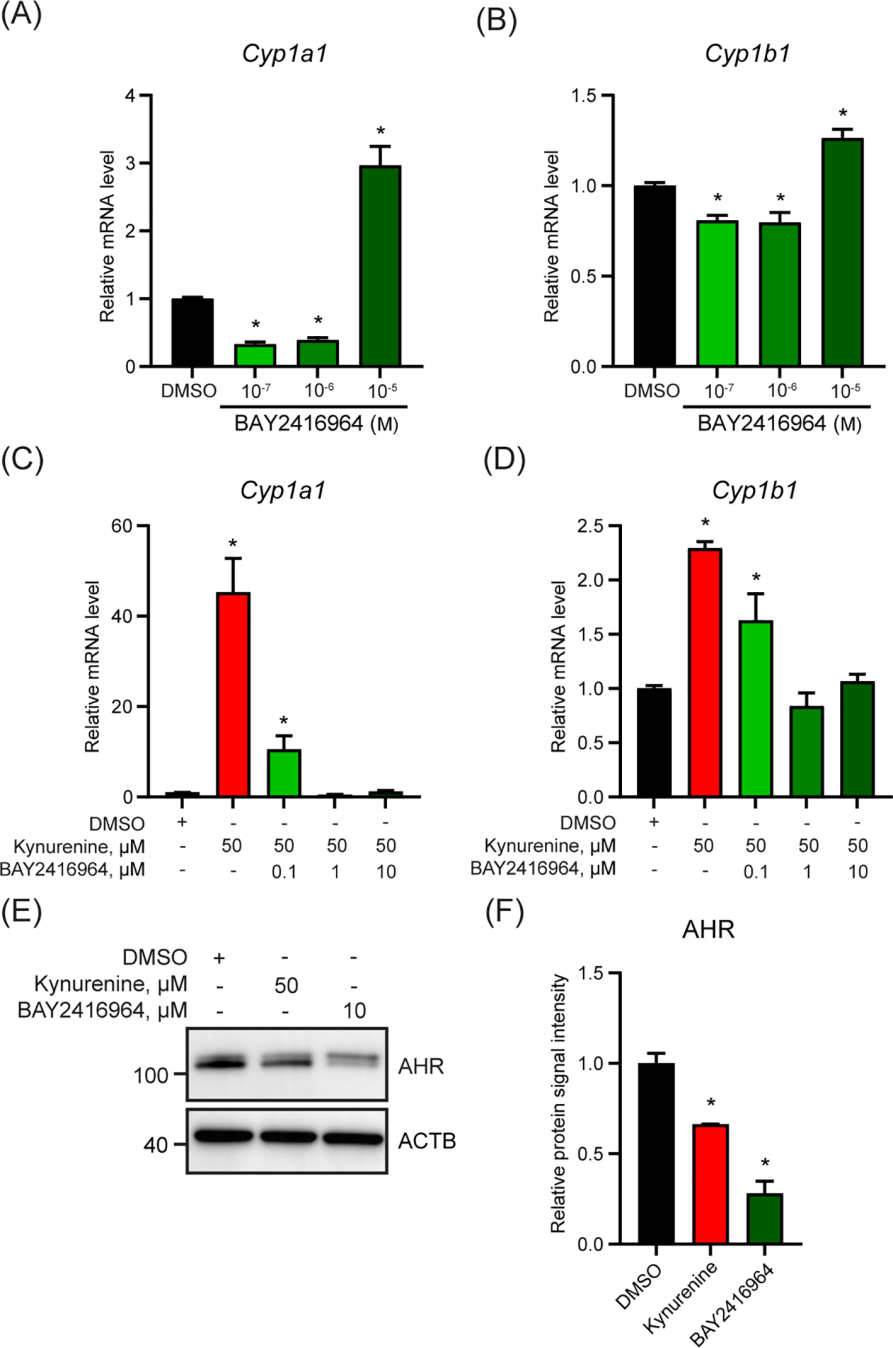
The murine pancreatic cancer cell line CR705 responds to BAY2416964 similarly as PyMT cells. Basel levels of *Cyp1a1* **(A)** and *Cyp1b1* **(B)** mRNA level decreased with 0.1 µM and 1 µM BAY2416964 and increased with 10 µM BAY2416964 in CR705 cells treated for 6 h. RT-qPCR of *Cyp1a1* **(C)** and *Cyp1b1* **(D)** mRNA levels in CR705 cells treated 6 h with kynurenine alone, or in combination with increasing doses of BAY2416964. RT-qPCR data presented as mean ± S.E.M. n=3 p<0.05. **(E)** Western blot of CR705 cells treated with 50 µM kynurenine or 10 µM BAY2416964. Representative image n=2. **(F)** Protein quantification of western blot presented in **(E)**. *p<0.05 compared with control (DMSO).

To evaluate if the observed effects of BAY2416964 were limited to PyMT cells, we examined the ability of BAY2416964 to inhibit AHR signaling in the murine pancreatic ductal adenocarcinoma cell line CR705. Treatment of CR705 cells with 0.1 and 1 µM reduced *Cyp1a1* and *Cyp1b1* levels compared with DMSO (**Figures 2A, B**). Similar to that observed for PyMT cells, 10 µM BAY2416964 also increased *Cyp1a1* and *Cyp1b1* levels in CR705 cells. Concentrations of 1 µM and 10 µM BAY2416964 were more effective than 0.1 µM BAY2416964 at inhibiting kynurenine-induced increases in *Cyp1a1* and *Cyp1b1* expression (**Figures 2C, D**). AHR protein levels were also reduced by 10 µM BAY2416964 (**Figures 2E, F**). Together, these data show that BAY2416964 and GNF351 antagonize ligand activated AHR, but also that high concentration of BAY2416964 destabilizes the AHR protein. Because of this, we used a concentration of 1 µM of BAY2416964 for the other studies in this work.

### 3.2 Loss of AHR and long-term exposure to BAY2416964, but not short-term, reduce PyMT cell proliferation and migration

**Figure 3:**
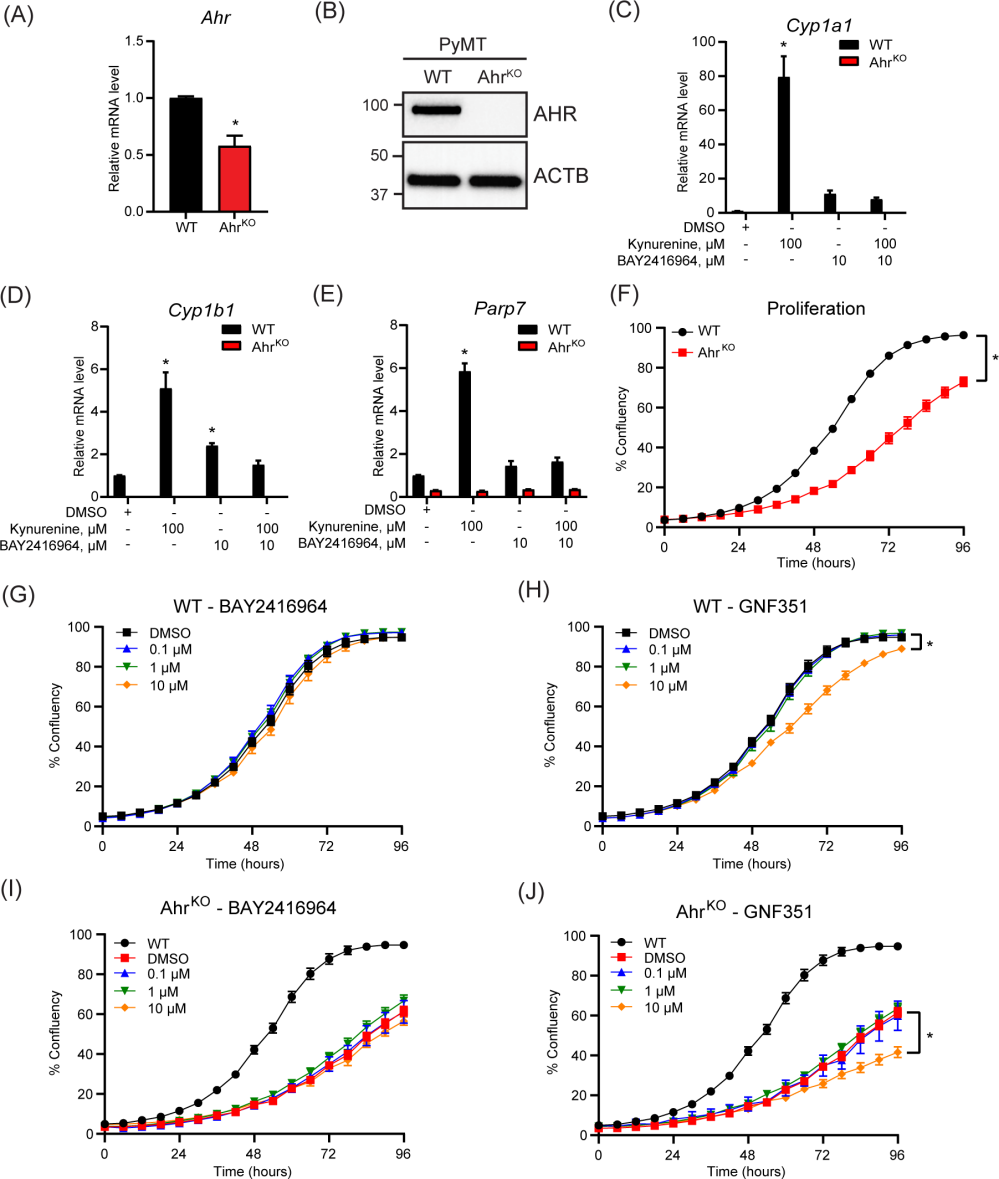
Loss of AHR prevents increases in kynurenine-induced AHR target genes and reduces proliferation of PyMT cells. **(A)** *Ahr* mRNA levels in PyMT Ahr^KO^ cells compared with WT cells as determined by RT-qPCR. Significance was determined with Student’s t-test, n=3 **(B)** Western blot of WT and Ahr^KO^ cells detected no AHR protein in the PyMT Ahr^KO^ cell line. Representative image of n=3. AHR activity was determined in the PyMT WT and Ahr^KO^ cell lines by treatment with 100 µM kynurenine, 10 µM BAY2416964 or combination for 6 hours. Kynurenine and high concentration of BAY2416964 failed to increase *Cyp1a1* **(C)**, *Cyp1b1* **(D)** and *Parp7* **(E)** expression levels in Ahr^KO^ cells. RT-qPCR results are presented as mean ± S.E.M. of n=3. Significance was determined using Two-Way ANOVA, p<0.05. * Significant from PyMT WT control (DMSO). **(F)** Ahr^KO^ cells proliferate more slowly than WT cells. **(G)** BAY2416964 did not affect proliferation of PyMT WT cells. **(H)** Proliferation of PyMT WT cells treated with increasing doses of GNF351. Treatment with 10 µM GNF351 decreased proliferation of PyMT WT cells. Proliferation of Ahr^KO^ cells treated with increasing concentrations of BAY2416964 **(I)** or GNF351 **(J)**. Only 10 µM GNF351 affected proliferation of Ahr^KO^ cells compared to DMSO control. WT was added for comparison. Data are presented as mean ± S.E.M of n=12, measured by IncuCyte. Significance was determined using Area under curve with p<0.05.

To compare the effect of pharmacological inhibition of AHR by BAY2416964 with the loss of AHR expression, we aimed to create Ahr^KO^ cells using CRISPR/Cas9. However, after selection, only one Ahr^KO^ cell line clone displayed the presence of indels in *Ahr* exon 1, resulting in frameshift mutations and premature stop codons determined by DNA sequencing (**Supplementary Figure S2**). AHR expression and activity was then confirmed by gene expression analysis and western blotting. PyMT Ahr^KO^ cells expressed less *Ahr* mRNA level than WT cells (**Figure 3A**), and no AHR protein was detected in the Ahr^KO^ cell line (**Figure 3B**). As expected, kynurenine increased *Cyp1a1*, *Cyp1b1* and *Parp7* mRNA levels in WT cells, but not in Ahr^KO^ cells (**Figures 3C–E**). We did not observe any induction of *Cyp1a1*, *Cyp1b1* or *Parp7* levels in the Ahr^KO^ cell line in response to 10 µM BAY2416964, showing that this effect is AHR dependent.

Since AHR affects cell proliferation through regulation of cell cycle and growth factors (4, 32), we investigated the proliferative effect of AHR inhibition. Loss of AHR significantly reduced proliferation of the Ahr^KO^ cells (**Figure 3F**). However, a four-day exposure of different concentrations of BAY2416964 did not affect cell proliferation, while 10 µM GNF351 significantly decreased the proliferation of PyMT WT cells (**Figures 3G, H**). BAY2416964 did not affect the proliferation of Ahr^KO^ cells (**Figure 3I**). Consistent with observations of WT cells, 10 µM GNF351 significantly decreased the proliferation of Ahr^KO^ cells (**Figure 3J**). These data suggest that the anti-proliferative effects of 10 µM GNF351 are AHR independent. Our findings also show that short-term treatment of BAY2416964 does not reflect the effect of loss of AHR in this cell line.

**Figure 4:**
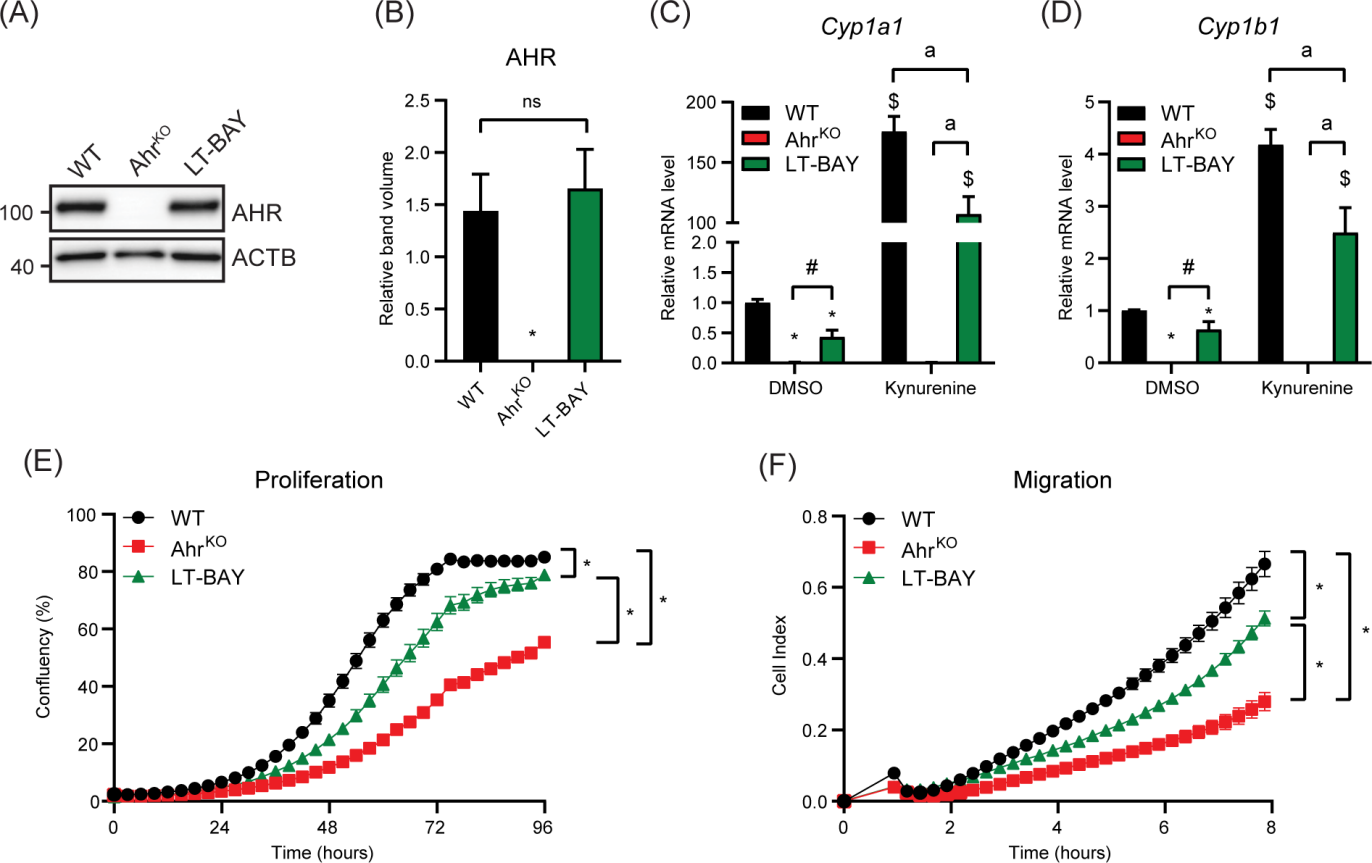
Characterization of Ahr^KO^ and long-term BAY2416964 treated PyMT cells. **(A)** Western blot of PyMT WT, PyMT Ahr^KO^ and PyMT LT-BAY cells show AHR protein levels are not changed in PyMT LT-BAY cells compared to WT cells. Representative image of n=3. **(B)** Quantification of western blot from figure **(A)**, presented as mean ± S.E.M of n=3. Relative *Cyp1a1* **(C)** and *Cyp1b1* **(D)** mRNA levels in PyMT WT, Ahr^KO^ and LT-BAY cells after exposure to 100 µM kynurenine for 6 hours, measured by RT-qPCR. Data are presented as mean ± S.E.M of n=3. *p<0.05 relative to WT DMSO, and ^#^p<0.05 between Ahr^KO^ and LT-BAY determined by one-way ANOVA for DMSO samples only. ^$^p<0.05 compared with WT DMSO and ^a^p<0.05 significance between indicated comparisons determined by two-way ANOVA. **(E)** Proliferation of PyMT WT, Ahr^KO^ and LT-BAY cells measured by IncuCyte. Data are presented as mean ± S.E.M. of n=16. **(F)** Migration of WT, Ahr^KO^ and LT-BAY cells in a transwell assay measured by xCELLigence. Data are presented as mean ± S.E.M. of n=8. Significance was determined by area under curve, p<0.05.

Since short-term treatment with BAY2416964 did not recapitulate the reduced proliferation observed in cells devoid of AHR, we examined whether long-term exposure to 1 µM BAY2416964 would better mimic loss of AHR protein. To this end, we exposed PyMT cells to 1 µM BAY2416964 for at least 6 weeks, which are hereafter referred to as LT-BAY cells. In contrast to that observed after 6 h exposure to BAY2416964 (**Figure 1L**), AHR protein levels were similar in LT-BAY cells compared with WT cells (**Figures 4A, B**); however, the constitutive levels of *Cyp1a1* and *Cyp1b1* were reduced in LT-BAY cells (**Figures 4C, D**). Kynurenine treated LT-BAY cells had reduced *Cyp1a1* and *Cyp1b1* mRNA levels compared with WT cells, but significantly higher levels than those observed in Ahr^KO^ cells. We next investigated the proliferative and migratory characteristics of LT-BAY cells. LT-BAY cells proliferated slower than WT cells, although not to the same extent as that observed in Ahr^KO^ cells (**Figure 4E**). We also observed reduced migration of Ahr^KO^ and LT-BAY cells compared with WT cells (**Figure 4F**).

### 3.3 Gene expression profiling identifies differential expressed genes that are commonly changed after AHR knockout or long-term BAY2416964 treatment

**Figure 5:**
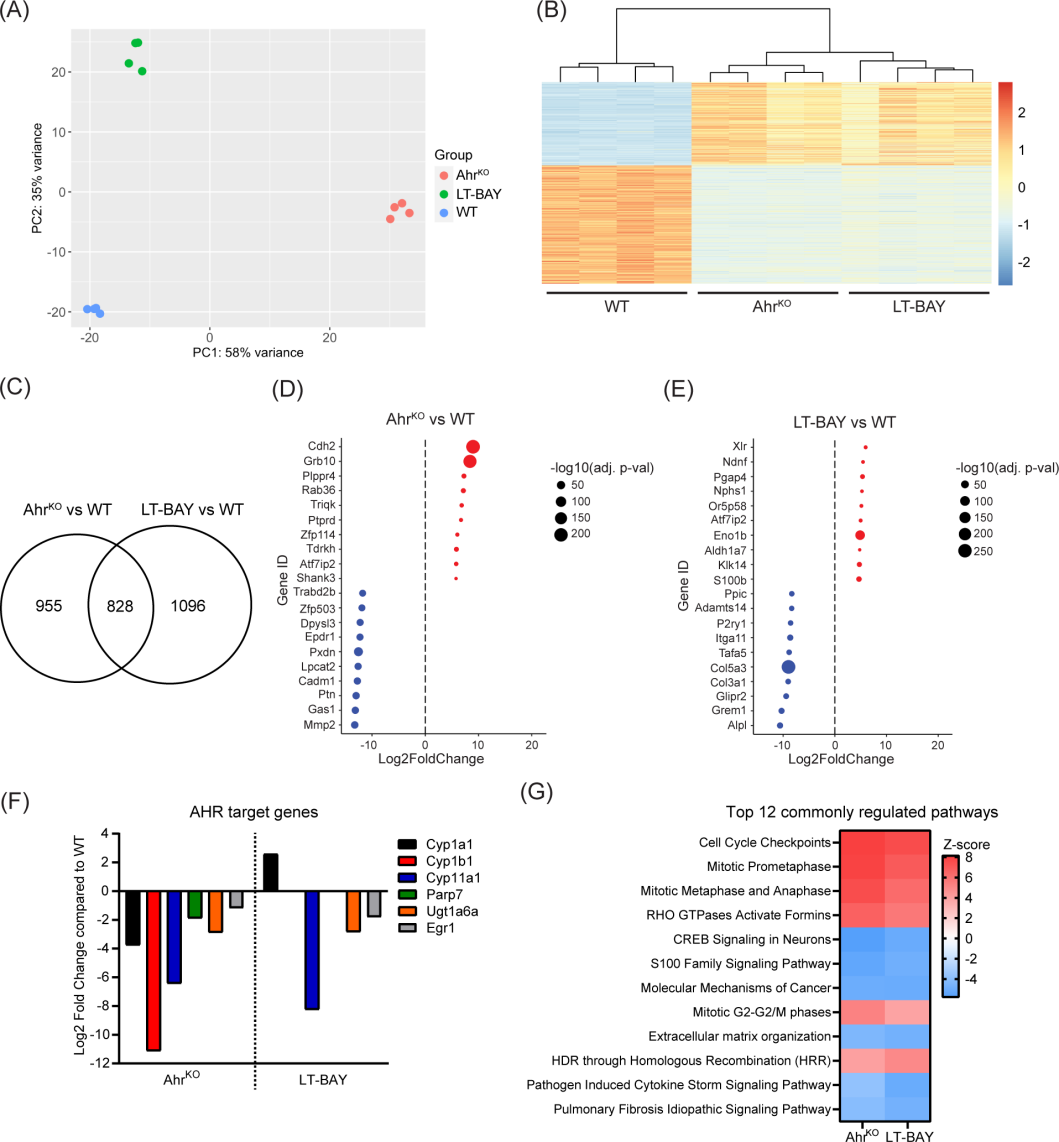
RNA sequencing revealing expressional effects of AHR inhibition. mRNA from PyMT WT, Ahr^KO^ and LT-BAY cells was sequenced and analyzed of n=4, p<0.01. **(A)** Principal component analysis (PCA) plot of the 4 replicates. **(B)** Heatmap illustrating the relative gene expression profiles of overlapping differentially expressed genes in Ahr^KO^ and LT-BAY cells relative to WT for each replicate. There is close homogeneity among the replicates, in addition to a greater effect of the genetic loss of *Ahr*, compared to pharmacological inhibition with BAY2416964. **(C)** Venn diagram of significantly changed genes in Ahr^KO^ or LT-BAY cells compared with WT. Top up- and downregulated genes in Ahr^KO^ **(D)** and in LT-BAY **(E)** compared with WT. Upregulated genes are marked in red, and downregulated genes in blue, with the size of the dot indicating the level of significance. **(F)** Presentation of AHR target genes that are significantly different in Ahr^KO^ compared to WT, and in LT-BAY compared to WT. **(G)** Comparison of the top 12 commonly regulated pathways in Ahr^KO^ or LT-BAY *vs* WT identified by ingenuity pathway analysis. Significantly changed pathways are defined by a Z-score >2 or <-2.

To identify differentially expressed genes (DEGs) among WT, Ahr^KO^ and LT-BAY cells, we performed RNA sequencing. We initially included WT cells exposed to 1 µM BAY2416964 for 24 h, but this did not result in any significant differences (absolute log2 fold change = 1) in gene expression compared with DMSO using p<0.01. Using a more relaxed statistical cutoff of p<0.05, we only identified two genes that were significantly upregulated, *eukaryotic translation initiation factor 4A pseudogene 4* (*Eif4a-ps4*) and *THAP domain containing 6* (*Thap6*), a gene that encodes a transcription factor in humans, but is characterized as a pseudogene in mice (33). Based on these findings, we focused on comparing WT, Ahr^KO^ and LT-BAY cells. Principal component analysis revealed distinct clustering among all three cell lines (**Figure 5A**). Hierarchical clustering using overlapping DEGs revealed similar expression patterns for overlapping DEGs between Ahr^KO^ and LT-BAY cells compared with WT (**Figure 5B**). We identified 1783 genes that were significantly changed in Ahr^KO^ cells, and 1924 genes that were changed in the LT-BAY cells compared with WT. Of these, 828 (46.4%) genes overlapped between Ahr^KO^ and LT-BAY cells (**Figure 5C**; **Supplementary Tables S1**, **S2**). A summary of the top 10 down- and upregulated genes in Ahr^KO^, or LT-BAY compared with WT, is provided in **Figures 5D, E**. Even though there were differences in gene expression profiles between Ahr^KO^ and LT-BAY cells, we observed a relatively high degree of overlapping genes between the two cell lines, suggesting that LT-BAY cells mimic many of the transcriptional changes induced by AHR knockout.

When we examined the expression levels of known AHR target genes, we observed that loss of AHR resulted in significant downregulation of the constitutive levels of AHR target genes such as *Cyp1a1*, *Cyp1b1*, *Cyp11a1*, *Parp7*, *UDP glucuronosyltransferase family 1 member A6* (*Ugt1a6*) and *early growth response 1* (*Egr1*). *Cyp11a1*, *Ugt1a6* and *Egr1* was also downregulated in LT-BAY cells. In contrast to the Ahr^KO^ cells, we did not observe a significant difference in *Cyp1b1* and *Parp7* levels, and surprisingly *Cyp1a1* levels were increased in LT-BAY cells (**Figure 5F**).

Ingenuity pathway analysis of the genes significantly changed in both Ahr^KO^ and LT-BAY cells was used to identify cellular pathways that were commonly changed in Ahr^KO^ and LT-BAY cells. The top 12 pathways are shown in **Figure 5G**. Commonly regulated pathways that were increased included cell cycle control, mitosis, RHO signaling, and homology-directed repair through homologous recombination (**Figure 5G**). Commonly regulated pathways that were decreased pathways included CREB signaling, S100 family signaling, molecular mechanisms of cancer and extracellular matrix organization. Notably, AHR signaling was increased in both Ahr^KO^ and LT-BAY cells, but this was not within the top 100 pathways identified. Although xenobiotic metabolism AHR signaling pathway was also not among the top 100 pathways, it was decreased in Ahr^KO^ cells but slightly increased in LT-BAY cells. The observed upregulation in these two pathways might be a result of compensation mechanisms due to the inhibition/loss of AHR or because prolonged exposure to 1 µM BAY2416964 might induce weak partial agonist activity like that observed for short term 10 µM BAY2416964. A complete list of the commonly regulated pathways is provided in **Supplementary Table S3**. Despite some differences, the gene expression findings reveal that long-term BAY2416964 treatment better mimics the effects observed after AHR loss than short term treatment.

### 3.4 Ectopic expression of AHR confirms the role of AHR in the regulation of *Cdh2, Grb10, Mmp2* and *Gas1* and reveals differential regulation in Ahr^KO^ compared with LT-BAY cells

**Figure 6:**
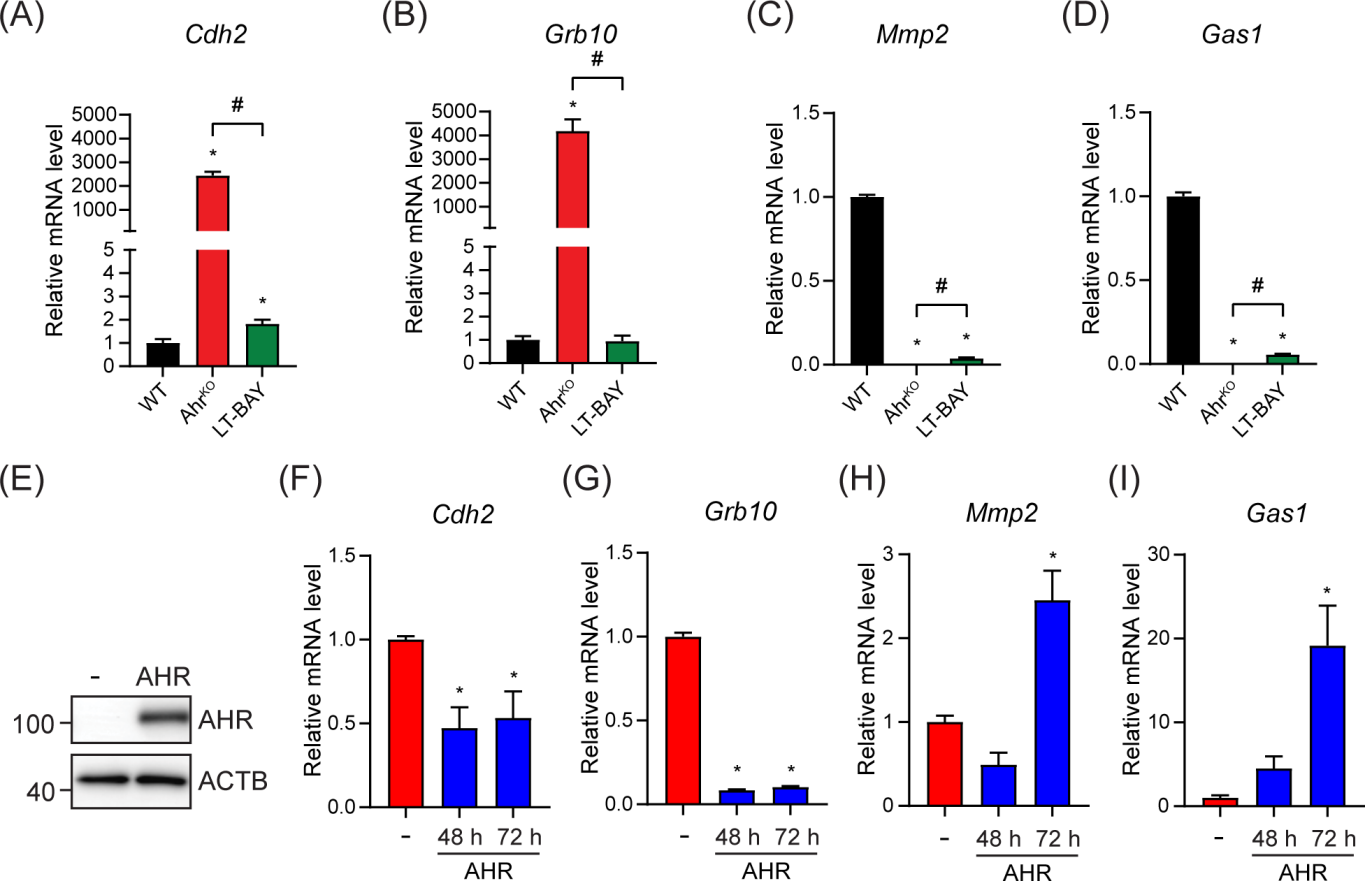
Ectopic AHR expression partially rescued the top up- and downregulated genes in the PyMT Ahr^KO^ cell line. The top two up- and downregulated genes in Ahr^KO^ cells were tested by RT-qPCR in WT, Ahr^KO^ and LT-BAY cells. **(A)** *Cdh2* was strongly upregulated in PyMT Ahr^KO^ cells, and slightly upregulated in LT-BAY cells compared with WT. **(B)** *Grb10* was upregulated in Ahr^KO^ cells, but not in LT-BAY cells. Both *Mmp2* **(C)** and *Gas1* **(D)** were downregulated in Ahr^KO^ and LT-BAY cells compared to WT cells. Data are presented as mean ± S.E.M. of n=3. **(E)** Western blot of Ahr^KO^ cells transfected with empty vector **(-)** or pcDNA3.1-Ahr. Representative image of n=3. *Cdh2* **(F)** and *Grb10* **(G)** mRNA levels were decreased after overexpression of AHR compared with empty plasmid at both time points. *Mmp2* **(H)** and *Gas1* **(I)** mRNA levels were increased 72 h after overexpression of AHR. Data are presented as mean ± S.E.M. of n=3, p<0.05.

We next used RT-qPCR to confirm the expression of the top two upregulated genes, *cadherin-2* (*Cdh2*) and *growth factor receptor-bound protein 10* (*Grb10*), and the top two downregulated genes, *growth arrest specific protein 1* (*Gas1*) and *matrix metalloproteinase 2* (*Mmp2*), in the Ahr^KO^ *vs* WT comparison. *Cdh2* codes for N-cadherin, which plays an important role in epithelial-mesenchymal transition (34). GRB10 is an adaptor protein associated with tyrosine kinase receptors and have been linked to increased tumor growth of different cancer types (35, 36). GAS1 causes cell cycle arrest in the G_0_ phase, thus inhibiting proliferation (37). MMP2 aids in degradation of extracellular matrix and enables invasion (34). In agreement with the RNA-sequencing data, *Cdh2* and *Grb10* mRNA levels were upregulated in PyMT Ahr^KO^ cells compared to WT. In LT-BAY cells, *Cdh2* was slightly increased compared with WT, but no changes were observed for *Grb10*. (**Figures 6A, B**). In contrast to the RNA-sequencing data, *Cdh2* was not among the DEGs in LT-BAY cells and *Grb10* was downregulated compared with WT. *Gas1* and *Mmp2* mRNA levels were downregulated in both Ahr^KO^ and LT-BAY cells compared with WT, supporting the RNA-sequencing results (**Figures 6C, D**). Because the observed upregulation of *Cdh2* and *Grb10*, and downregulation of *Gas1* would suggest a more protumorigenic phenotype with increased migration, we looked at the expression levels of several epithelial-mesenchymal transition (EMT) related genes (34). We found that the majority of EMT related genes were downregulated in both Ahr^KO^ and LT-BAY cells compared with WT (**Supplementary Figures S3A, B**). This suggests that even though some genes that promote EMT were increased in Ahr^KO^ and LT-BAY cells, most of the genes associated with EMT were reduced, supporting the reduced migration of Ahr^KO^ and LT-BAY cells compared to WT cells.

To establish the role of AHR in the regulation of *Cdh2*, *Grb10*, *Gas1* and *Mmp2* we overexpressed mouse AHR in Ahr^KO^ cells. Western blots confirmed the expression of AHR (**Figure 6E**). Ectopic AHR expression significantly decreased *Cdh2* and *Grb10* mRNA levels after both 48 h and 72 h compared with empty vector controls (**Figures 6F, G**). AHR overexpression increased *Gas1* and *Mmp2* mRNA levels after 72 h (**Figures 6H, I**). To determine if *Cdh2*, *Grb10*, *Gas1* and *Mmp2* contained AHREs, we extracted the genomic regions ±1000 bp from the transcriptional start sites (TSS) of each gene and probed them for AHR binding motifs using JASPAR (38). We identified multiple putative AHREs in all sequences (**Supplementary Table S4**). However, additional studies will be needed to confirm whether the putative AHREs are bound by AHR and functional. Taken together, these results suggest that the observed transcriptional changes in Ahr^KO^ cells are due to the loss of *Ahr* and not off target effects of the CRISPR/Cas9, and that the regulation of *Cdh2*, *Grb10*, *Gas1* and *Mmp2* occurs through canonical AHR signaling; however, we can’t completely exclude that non-canonical AHR signaling might also contribute to this regulation.

## 4 Discussion

AHR is recognized as an attractive therapeutic target because of its central role in coordinating cancer cell responses to metabolic changes, genetic alterations, environmental signals and immune activities (8, 9). AHR is overexpressed in many cancer types and has been reported to exhibit diverse pro-survival functions. Many of these carcinogenic actions of AHR are driven by kynurenine and its derivatives. IDO1 and TDO2 generated kynurenine in cancer cells, increases kynurenine concentrations in tumors and plasma which is associated with poor prognosis, immunosuppression and tumor progression (39). Thus, inhibiting AHR activity represents an exciting therapeutic strategy to target multiple cancer dependencies by blocking kynurenine signaling downstream of IDO1 and TDO2. In support of this, IK-175 is being tested in a clinical trial as monotherapy against urothelial carcinoma or in combination with check point inhibitor, nivolumab (NCT04200963) (40), while BAY2416964, is being tested against advanced solid tumors alone or in combination with pembrolizumab (NCT04069026 and NCT04999202) (41, 42). BAY2416964 is a relatively new AHR antagonist and there are limited studies describing its ability to inhibit AHR and affect AHR dependent regulation of intrinsic cancer cell functions.

Here, we investigated the effects of short-term (<24 h) and long-term (>6 weeks) treatment with BAY2416964 and compared them with AHR loss in a PyMT mouse mammary tumor cell line. Because we observed that short-term BAY2416964 treatment did not reproduce many of the phenotypes we observed in Ahr^KO^ cells, we generated LT-BAY by culturing PyMT cells for at least 6 weeks in the presence of 1 μM BAY2416964. Since the generation of tamoxifen resistant breast cancer cell lines through continual exposure to tamoxifen have greatly increased our understanding of the mechanisms of resistance (43), the long-term treatment with BAY2416964 should also provide valuable insight into how extended treatment affects cancer cell signaling. Although kynurenine-induced AHR activity was inhibited in both short- and long-term BAY2416964, the reduced cell proliferation and migration characteristic of Ahr^KO^ cells was only observed in LT-BAY cells. Despite some differences in gene expression profiles between LT-BAY and Ahr^KO^ cells, 828 genes (46.4%) were common to both cell lines. This suggests that a prolonged exposure to BAY2416964 better mimics the effects of loss of AHR.

In line with BAY2416964 acting as an AHR antagonist (21), BAY2416964 effectively inhibited kynurenine-induced AHR target genes, including *Cyp1a1* and *Cyp1b1.* Lower increases in *Cyp1a1* and *Cyp1b1* levels in response to kynurenine were also observed in LT-BAY cells. Unlike GNF351, we observed significant increases in *Cyp1a1* and *Cyp1b1* after treatment with 10 μM BAY2416964 in both PyMT and CR705 cells. No increases were observed in PyMT Ahr^KO^ cells, showing that these increases were AHR dependent. RNA sequencing of LT-BAY cells revealed an increase in *Cyp1a1* expression, but not *Cyp1b1*. These results are in contrast with a previous report in which no AHR activation by BAY2416964 was observed in human U87 and immune cells as well as mouse Hepa1c1c7 cells (21). However, other AHR antagonists including α-naphthoflavone and 3´-methoxy-4´-nitroflavone have been reported to exhibit partial agonist activities in the absence of exogenous AHR ligands (44, 45). In addition, mexiletine exhibits AHR antagonist activity in human MDA-MB-468 breast cancer cells but acts as an AHR agonist in rat hepatoma cells (46, 47). Our findings and the above reports support that many AHR ligands exhibit selective agonist and antagonist activity that is cell-type and context-dependent (46, 48). GNF351 did not affect AHR protein levels while BAY2416964 reduced total and cytoplasmic AHR protein levels in a manner similar to that reported for IK-175 and CH223191 (22). The increased nuclear translocation of AHR, together with the increase of AHR target genes, supports a partial agonist effect of 10 μM BAY2416964. In contrast, no differences in AHR protein levels were observed in LT-BAY cells compared with WT cells, implying that BAY2416964 induces only a transient decrease in AHR protein levels that is not apparent after prolonged treatment. Our findings suggest that treatment with BAY2416964 alone induces cell-type and context dependent increases of CYP1A1; however, this will need to be tested in additional cell lines and across different species.

AHR regulates cancer cell proliferation and migration (4, 48), but the anti-proliferative and anti-migratory effects of BAY2416964 have not been previously described (21). The reduced proliferation and migration we observed in Ahr^KO^ cells compared to WT cells was similar to the reduced proliferation reported in Ahr^KO^ human colon cancer cell lines (49). Several AHR antagonists have also been reported to reduce cancer cell proliferation and migrations, including CH223191, 6,2´,4´-trimethoxyflavone and GNF351 (50, 51). GNF351, but not BAY2416964, reduced proliferation of both PyMT and Ahr^KO^ cells, suggesting that the antiproliferative effects of GNF351 on PyMT cells are independent of AHR. However, LT-BAY cells exhibited reduced proliferation and migration compared with WT cells, confirming that like other AHR antagonists, BAY2416964 also reduced cancer cell proliferation and migration, but only after long-term treatment.

Because we only observed 2 DEGs, both of which are pseudogenes, after 24 h treatment with 1 μM BAY2416964, we compared the gene expression profiles of LT-BAY with those of Ahr^KO^ cells. Indeed, LT-BAY cells better reflect what was observed in Ahr^KO^ cells, suggesting a BAY2416964-dependent dampening of AHR signaling over time. This was observed in cell proliferation, migration and the relatively high degree of overlap in DEGs, differentially regulated pathways and hierarchical clustering between Ahr^KO^ and LT-BAY cells. There were also many DEGs that were unique to either Ahr^KO^ or LT-BAY cells, which could reflect AHR-independent effects of BAY2416964. Although the addition of AHR into Ahr^KO^ cells reversed the expression levels of the genes tested, we cannot exclude the possibility that some of the DEGs identified or the phenotypic changes in the LT-BAY and Ahr^KO^ cells could be due to adaptation to AHR inhibition or off target effects of CRISPR/Cas9 rather than loss of AHR activity. However, due to difficulties in generating the Ahr^KO^ cell line, we were only successful at isolating one clone.

Overall, we confirm that BAY2416964 is a potent AHR antagonist and inhibits kynurenine-induced AHR activation. We show that prolonged pharmacological inhibition of AHR with BAY2416964 mimics many of the phenotypes observed after AHR loss, including reduced cell proliferation and migration. Our findings further support targeting AHR with BAY2416964 as an innovative cancer treatment strategy.

## Data Availability

The datasets presented in this study can be found in online repositories. The names of the repository/repositories and accession number(s) can be found below: https://www.ncbi.nlm.nih.gov/geo/, GSE272215.
