# Corrigendum: Long-term exposure to BAY2416964 reduces proliferation, migration and recapitulates transcriptional changes induced by AHR loss in PyMT-induced mammary tumor cells

**DOI:** 10.3389/fonc.2024.1516633

**Published:** 2024-11-19

**Authors:** Ninni Elise Olafsen, Siddhartha Das, Chiara Gorrini, Jason Matthews

**Affiliations:** ^1^ Department of Nutrition, Institute of Basic Medical Sciences, Faculty of Medicine, University of Oslo, Oslo, Norway; ^2^ School of Molecular and Cellular Biology, University of Leeds, Leeds, United Kingdom; ^3^ Princess Margaret Cancer Centre, University Health Network, Toronto, ON, Canada; ^4^ Department of Pharmacology and Toxicology, University of Toronto, Toronto, ON, Canada

**Keywords:** aryl hydrocarbon receptor, BAY2416964, breast cancer, PyMT, GNF351, proliferation, kynurenine

In the published article, there was an error. The the guide oligo sequences describing the gRNA used to target *Ahr* were incorrect.

A correction has been made to **Methods and materials,** *2.3 Generation of the PyMT Ahr^KO^ cell line*. This sentence previously stated:

“Briefly, the guide oligos forward 5´-CCTACGCCAGTCGCAAGCGG-3´ and reverse 5´-CCGCTTGCGACTGGCGTAGG-3´ were annealed and ligated into the pSpCas9(BB)-2A-Puro (PX459) plasmid (Addgene, Watertown, MA, USA; plasmid #62988).”

The corrected sentence appears below:

“Briefly, the guide oligos forward 5´-AAACTCTAAGCGACACAGAGACCGC-3´ and reverse 5´-CACCGCGGTCTCTGTGTCGCTTAGA-3´ were annealed and ligated into the pSpCas9(BB)-2A-Puro (PX459) plasmid (Addgene, Watertown, MA, USA; plasmid #62988).”

The authors apologize for this error and state that this does not change the scientific conclusions of the article in any way. The original article has been updated.

