# Supplementary material for: Long-term exposure to BAY2416964 reduces proliferation, migration and recapitulates transcriptional changes induced by AHR loss in PyMT-induced mammary tumor cells
Source: Front Oncol. 2024 Oct 10;14:1466658. doi: 10.3389/fonc.2024.1466658 (PMC11499230; doi:10.3389/fonc.2024.1466658)
Supplement: Supplementary file 1 [file Image1.pdf]

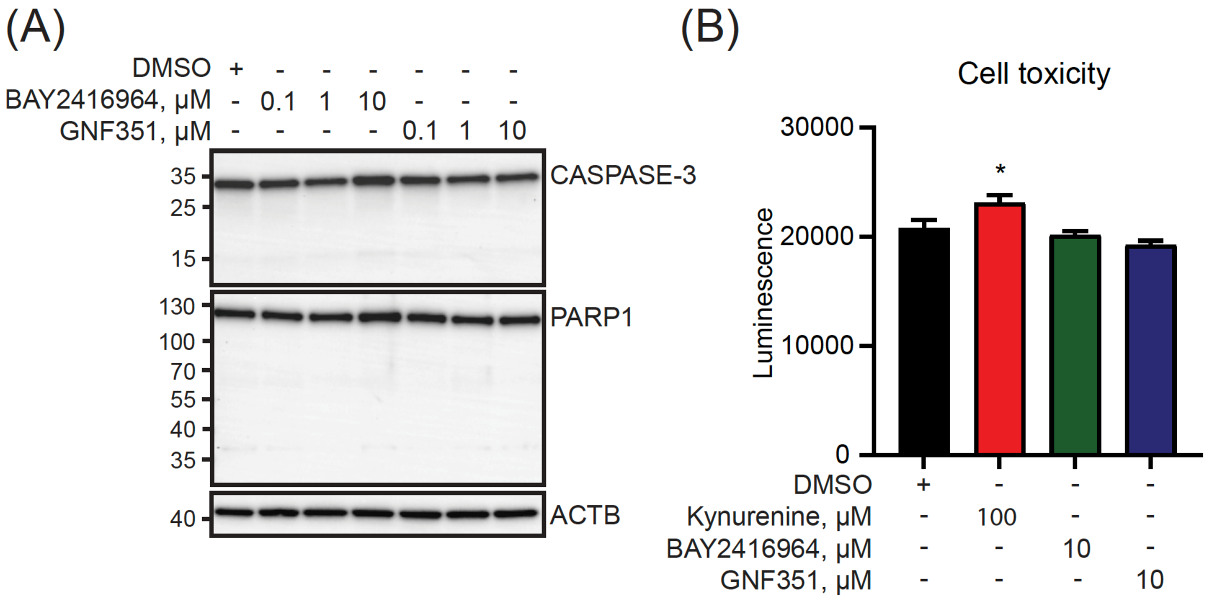

Supplementary Figure S1. High concentrations of BAY2416964 or GNF351 do not induce apoptosis in PyMT cells. (**A**) Western blot of PyMT cells treated 6 h with BAY2416964 or GNF351 at increasing concentrations reveal no Caspase3- or PARP1-cleavage. Overexposed representative image of n=3. (**B**) Treatment for 6 h with 100 µM kynurenine, or 10 µM with BAY2416964 or GNF351 were not toxic to the PyMT cells. Measure by CellTiter Glo assay, and presented as mean ± S.E.M. of n=6.*p<0.05.
