# Supplementary material for: Long-term exposure to BAY2416964 reduces proliferation, migration and recapitulates transcriptional changes induced by AHR loss in PyMT-induced mammary tumor cells
Source: Front Oncol. 2024 Oct 10;14:1466658. doi: 10.3389/fonc.2024.1466658 (PMC11499230; doi:10.3389/fonc.2024.1466658)
Supplement: Supplementary file 2 [file Image2.pdf]

```
Wildtype
 A  E  G  I  K  S  N  P  S  K  R  H  R  D  R  L  N  T  E  L
GCTGAAGGAATTAAGTCAAATCCTTCTAAGCGACACAGAGACCGGCTGAACACAGAGTTA

AhrKO clone
GCTGAAGGAATTAAGTCAAATCCTTCT-AGCGACACAGAGACCGGCTGAACACAGAGTTA
GCTGAAGGAATTAAGTCAAATCCTTCT--GCGACACAGAGACCGGCTGAACACAGAGTTA
```

Supplementary Figure S2. DNA sequencing of the PyMT Ahr^{KO} clone revealed deletion of 1 or 2 base pairs, resulting in frameshift mutation and premature stop codons. Top strand shows DNA sequence of wildtype *Ahr* starting at alanine 27 in the protein sequence. Dashes (-) indicate base pair deletion.
