# Supplementary material for: Long-term exposure to BAY2416964 reduces proliferation, migration and recapitulates transcriptional changes induced by AHR loss in PyMT-induced mammary tumor cells
Source: Front Oncol. 2024 Oct 10;14:1466658. doi: 10.3389/fonc.2024.1466658 (PMC11499230; doi:10.3389/fonc.2024.1466658)
Supplement: Supplementary file 3 [file Image3.pdf]

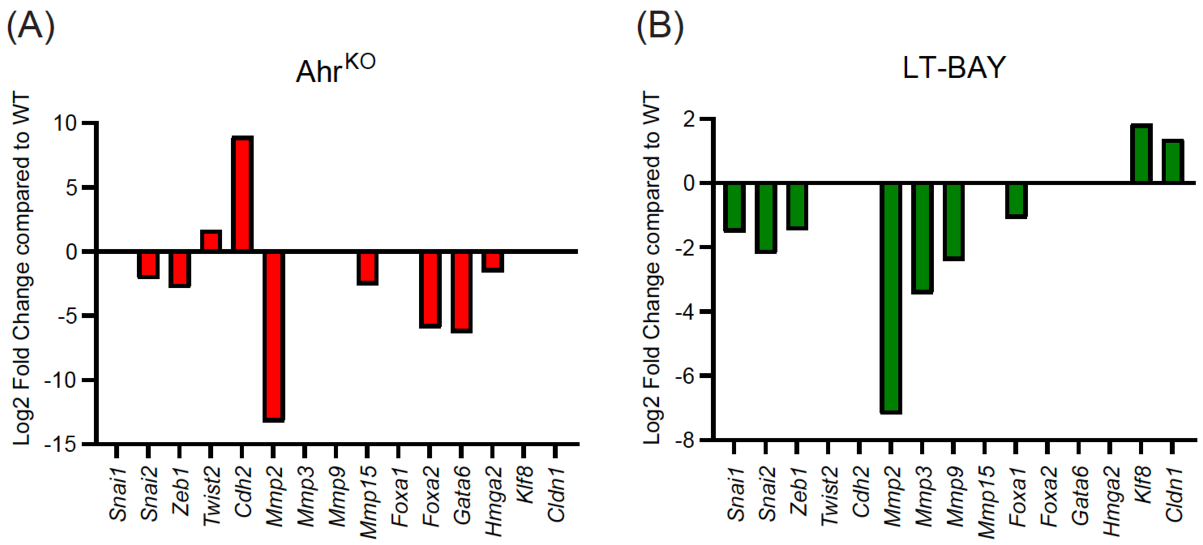

Supplementary Figure S3. Epithelial-mesenchymal transition (EMT) related genes from RNA sequencing. Genes significantly different in Ahr^{KO} (**A**) or LT-BAY (**B**) cells compared to WT, determined by adjusted *p*-value <0.01.
